# The Double-Edged Sword Effect of Government Chatbot Empathy on Citizens’ Continued Usage Intention

**DOI:** 10.3390/bs16071095

**Published:** 2026-07-02

**Authors:** Xuesong Li, Yangying Zhou, Muqun Hu

**Affiliations:** School of Public Administration, Northeast Agricultural University, Harbin 150006, China; lixs@neau.edu.cn (X.L.); a12240225@neau.edu.cn (M.H.)

**Keywords:** government chatbot, empathy, continued usage intention, psychological engagement, information richness, time pressure

## Abstract

With recent breakthroughs in affective computing and AI algorithms, government chatbots are evolving from function-oriented tools to emotionally responsive agents capable of detecting, decoding, and responding to citizens’ emotional cues. This shift holds significant potential for enhancing citizens’ continued usage intention. Drawing on social presence theory and employing a scenario-based experimental design, this study investigates whether, how, and under what conditions empathy by government chatbots affects citizens’ willingness to continue using such services. The findings reveal that empathy positively influence continued usage intention by enhancing users’ psychological engagement and perceived information richness. However, the strength of this effect is significantly moderated by the type of time pressure. Specifically, under endogenous time pressure, the positive effect of government chatbot empathy is amplified, whereas under exogenous time pressure, the effect is attenuated. This study uncovers the double-edged nature of empathic design in government chatbots, contributing to the literature on human–robot interaction in public service contexts. It also offers practical implications for the adaptive design of empathic government chatbots to optimize citizen engagement and service effectiveness.

## 1. Introduction

As the new wave of industrial transformation deepens, countries are leveraging AI to implement user-centered intelligent government transformation, with government chatbots serving as a prime example of this practice (Nielsen et al., 2016). Government chatbots refer to computer programs designed to interact with citizens through digital platforms, such as government websites and WeChat official accounts, to provide information, answer inquiries, and deliver public services (Whitford et al., 2020). Many government chatbots also integrate with departmental data interfaces, enabling seamless embedding of multi-departmental services. Users need only input relevant information and grant authorization within the service interface to receive instant delivery of government services (B. Wang & Niu, 2023). However, these chatbots have long been constrained by mechanical service delivery and highly scripted interactions, often perceived as lacking human-like attributes (Van Noordt & Misuraca, 2022). Yet breakthroughs in advanced algorithms like affective computing have endowed government chatbots with new social interaction skills—chatbot empathy. This refers to a chatbot’s ability to detect, decode, and mimic users’ emotions and attitudes while responding appropriately, thereby enabling chatbot to empathize with users (Juquelier et al., 2025). Chatbot empathy marks a new phase in human–robot interaction, evolving from mere information organization to machines conveying emotions to humans (Luo et al., 2025). As an emerging governmental tool, chatbots require governments to enhance citizens’ willingness to use them sustainably in order to maximize their service effectiveness. Chatbot empathy is considered a key factor in bridging the gap between humans and AI in terms of users’ continued usage intention (M. Li & Wang, 2023). Consequently, numerous local governments in China have deployed empathetic government chatbots tailored to their specific needs, such as Beijing’s “Jingjing” and Shanghai’s “Xiao i.”

Echoing the growing interest among practitioners in empathic chatbots, an expanding body of literature has investigated the role of empathy in service interactions between customers and chatbots. The literature can be organized into two primary research streams. The first stream explores how mere and latent perceptions of empathy triggered by mechanical chatbots shape various customer responses (G. L. Chen et al., 2023; Y. L. Liu et al., 2023). Drawing on advances in affective computing, the second stream treats artificial empathy as a core intelligent framework for chatbots, which has the potential to substantially transform communication dynamics and reshape customer experiences, thereby warranting greater scholarly attention (Liu-Thompkins et al., 2022). Grounded in social presence theory (Short et al., 1976), the present study aligns with this second stream and extends prior research in two key respects (see Table 1).

First, research on chatbot empathy primarily focuses on commercial chatbots, with insufficient attention given to government chatbots. Even within the commercial sphere, consensus remains elusive regarding the relationship between chatbot empathy and customer outcomes. On one hand, studies indicate that chatbot empathy enhances human–robot interaction, thereby improving customer experience. For instance, Shi et al. (2025) explored empathetic strategies of AI chatbots and revealed that such strategies could notably boost users’ psychological engagement, thereby driving prosocial behaviors and fostering more positive service evaluations. On the other hand, G. Park et al. (2023) demonstrated that in digital voice assistant usage scenarios, human-like features such as emotional interaction did not significantly influence user behavior and had limited effects. This suggests that chatbot empathy may exert a double-edged sword effect on users’ continued usage intention, an effect yet to be examined in the public service domain. In fact, compared to commercial chatbots, government chatbots possess unique characteristics in both service content and objectives. Citizens’ interactions with them involve not only emotional needs (e.g., psychological engagement) but also functional demands (e.g., information richness) regarding the provision of accurate and comprehensive information, which distinguishes public service chatbots from commercial ones. This aligns with the core tenets of social presence theory: individuals not only evaluate the psychological engagement elicited by interactions but also assess the richness of information conveyed during communication to achieve their goals (Ostrom et al., 2021). Within this theoretical framework, this study explores how government chatbot empathy influences citizens’ continued usage intention through two parallel mediating mechanisms: psychological engagement and information richness.

Second, existing research has begun to examine time constraints in human–robot interaction, revealing that users’ experiences with chatbots are often influenced by perceived time pressure (Ostrom et al., 2021; Roy & Naidoo, 2021). However, such studies fail to distinguish the sources of time pressure. In government service scenarios, citizens interacting with government chatbots may experience both objective time pressure (driven by exogenous factors like service timeliness and task urgency) and subjective time pressure (arising from endogenous psychological expectations such as personal efficiency preferences and pacing control). Therefore, this study adopts a source-based perspective to distinguish between endogenous and exogenous time pressure. It explores the moderating effects of these factors on how government chatbot empathy shapes citizens’ willingness to continue usage, thereby testing the “double-edged sword” effect inherent in such chatbot empathy.

Overall, the current findings suggest that chatbot empathy may function as a double-edged sword, shaping citizens’ continued usage intention through multiple pathways. These insights offer practical guidance for practitioners on whether and how to embed empathy into their AI-driven interfaces, with the goal of improving citizens’ continued usage intention and ultimately driving higher satisfaction.

## 2. Literature Review

### 2.1. Government Chatbot Empathy

Chatbots are conversational system interfaces based on AI that enable human–robot interaction through natural language, commonly used for information exchange and service processing in specific scenarios (Meng & Ju, 2021). Supported by algorithms such as machine learning and natural language processing, chatbots possess intelligent recognition and response capabilities, effectively meeting users’ personalized needs (B. Wang & Niu, 2023). By type, chatbots can be categorized into two main classes: mechanical chatbots and empathic chatbots (Nißen et al., 2022). The former excel at handling short-term, standardized routine tasks with cost-effectiveness, though they are often perceived as impersonal and lacking emotional responsiveness (Huang & Rust, 2021). The latter possess empathy capabilities, simulating human emotional reactions during interactions by identifying, decoding, and responding to users’ emotional states (Wahbi et al., 2023). Research indicates that endowing chatbots with empathy enhances user trust, satisfaction, and willingness for continued use (Y. Park et al., 2024; Trivedi, 2019). In reality, neither type of chatbot genuinely experiences emotions—they merely appear to have real emotions (Nißen et al., 2022). Nevertheless, this does not diminish empathy as a priority development direction for next-generation service chatbots (Lim & Okuno, 2015; J. Fan et al., 2023).

E-government is one of the primary domains benefiting from the deployment of service chatbots (B. Wang & Niu, 2023). E-government refers to government departments leveraging information and communication technologies to enhance public service quality and improve citizen welfare (Meng & Ju, 2021). AI has transformed how e-government interacts with citizens, with government chatbot playing a pivotal role in this shift. First, chatbots can assist with policy consultation. Empowered by natural language processing technologies, government chatbots can replace human civil servants in providing citizens with policy interpretations and business consulting services, thus cutting down communication costs. Second, they facilitate transaction processing. Integrating process automation and optical character recognition technologies, government chatbots are capable of assisting citizens in completing such formalities as document application and fee payment, streamlining the entire administrative workflow. Third, they enable data analysis. Government chatbots can interface with data aggregation platforms and electronic certificate repositories, enabling the integration, quantification and mining of various government data and thereby providing data-driven support for governmental decision-making (X. Li & Wang, 2024). For government departments, the deployment of chatbots is an inevitable trend in the intelligent transformation of government services and the construction of a citizen-satisfaction-oriented government. Given the functional advantages of government chatbots, how to drive citizens to accept such chatbots voluntarily and enhance their willingness to use them on a continued basis has become a critical issue that government departments need to address urgently, and chatbot empathy is recognized as the key to achieving this goal.

Among academic scholars, a variety of theoretical models have been adopted to examine users’ willingness to continue using AI, including the Computers are Social Actors Theory (CASA), Attribution theory, Unified Theory of Acceptance and Use of Technology, and Expectation Confirmation theory. All these existing models seek to identify the key factors that influence individuals’ continued usage of technological tools, and the majority of the scholarly literature exploring users’ willingness to keep using service robots is grounded in the aforementioned theoretical frameworks (Teng, 2013). However, such theoretical approaches only account for the internal mechanisms underlying users’ willingness to continue using AI from the perspective of relational needs, while neglecting their functional demands for AI. This functional aspect is particularly crucial for government chatbots. Therefore, it is necessary to analyze citizens’ willingness to continue using AI from both relational and functional dimensions. Based on this analysis, this study employs social presence theory to examine the influence mechanism of government chatbot empathy on citizens’ willingness to continue using them.

### 2.2. Social Presence Theory

In 1976, Short, Williams, and Christie first proposed the concept of “social presence” to explain how media convey verbal and nonverbal cues, thereby enabling users to perceive the establishment of interactive relationships with others (Teng, 2013). By the 1990s, this theory expanded into computer-mediated communication, describing individuals’ ability to establish a “sense of presence” with spatially separated others through computer media. The medium’s capacity to convey social cues—such as spatial distance, facial expressions, linguistic style, and gender—directly influences users’ experience of social presence. Moreover, information transmission achieves maximum effectiveness only when the richness of information provided by the medium aligns with the complexity of the interactive task (Dai & Lu, 2015). Social presence theory reveals how media technology enhances social presence by fulfilling users’ relational and functional needs, thereby elevating positive psychological perceptions (Mao & Yuan, 2018). Social presence can be measured across four dimensions: Psychological Engagement, Copresence, Intimacy, and Behavioral Engagement. To be specific, psychological engagement denotes the authentic sense of face-to-face interaction that users gain during communication, through which they perceive others’ support and thus form genuine social interaction experiences. Copresence reflects the extent to which individuals feel the presence, companionship and interaction of others. Intimacy highlights the open, close and stable emotional bonds established in the course of communication. Behavioral engagement reflects users’ subjective assessments of the presence of dependence, connection, or interactive responses between their own behaviors and those of others (Short et al., 1976). Based upon these four dimensions, Salinäs (2002) extended a new dimension—information richness. Defined as individuals’ evaluation of the effectiveness and completeness of information and knowledge transmitted by media, this dimension underscores the functional orientation of individuals’ rational cognition and social presence. Current research in marketing and strategic management generally adopts these five dimensions to explore users’ psychological responses to computer-mediated platforms (e.g., internet platforms, live streaming, television advertisements) (G. Li et al., 2024; Ngo, 2025). However, empirical research applying social presence theory to human–robot interaction remains limited. For instance, studies by Juquelier et al. (2025), M. Kim (2024), and J. Chen et al. (2025) found that among the five dimensions, only psychological engagement and information richness were associated with users’ perceptions of human–robot interaction. Based on this, this study posits that psychological engagement and information richness still significantly influence citizens’ continued usage intention of government chatbot empathy.

Next, we present our theoretical framework outlining how government chatbot empathy affects citizens’ continued usage intention, with psychological engagement and perceived information richness serving as mediating mechanisms and time pressure acting as a moderating factor. Given the communicative nature of interactions between chatbots and citizens, our hypotheses draw on the communication science literature and social presence theory. Social presence theory is widely recognized as a foundational communication theory (Moffett et al., 2021) and has recently emerged as one of the most influential theoretical lenses in service robot research (Mariani et al., 2023). Originally developed within communication science to explain how communication media convey nonverbal and social cues (Short et al., 1976), the theory has subsequently underpinned research on computer-mediated communication, particularly concerning how a user’s “sense of being with another” and perceptions of information richness are shaped by various interfaces, content elements, and contextual factors (Biocca et al., 2003; Straub & Karahanna, 1998).

### 2.3. The Impact of Government Chatbot Empathy on Citizens’ Continued Usage Intention

Continued usage intention is a significant concept proposed in information systems research, describing users’ subjective willingness to continue using a particular information system over an extended period (J. Chen et al., 2025). M. Li and Wang (2023) further identified continued usage intention as a key indicator for measuring user loyalty and stickiness. Empirical research has shown that the cost of acquiring new users is approximately five times that of retaining existing ones (Spreng et al., 1995). Consequently, continued usage intention also serves as a core metric requiring urgent attention during the design and deployment of government chatbots by e-government departments (Whitford et al., 2020).

In interpersonal interactions, empathy refers to an individual’s appropriate response based on accurately perceiving another’s emotions and considering the specific context. This not only demonstrates understanding of another’s situation but also involves active cognitive engagement (Lim & Okuno, 2015). Therefore, service chatbots powered by natural language processing technology are often regarded as a significant barrier to user satisfaction when simulating human interactions, primarily due to their lack of genuine empathy capabilities (Shi et al., 2025). Lim and Okuno (2015) have demonstrated that users often experience a sense of monotony when interacting with mechanized chatbots devoid of emotional feedback, a perception that stands in stark contrast to the engaging experience offered by human civil servants. In the realm of government services, empathy serves as a vital communication tool for frontline civil servants, manifesting in service behaviors such as “enthusiastic initiative” and “patient attention to detail.” These behaviors not only influence citizens’ subjective evaluations of service quality but also directly impact the effectiveness of public service outcomes (X. Li & Wang, 2024). In traditional public service delivery, when citizens explicitly express their “empathy needs” during interactions, a lack of responsive empathy by service providers may trigger negative emotions such as misunderstanding, hostility, and frustration among citizens, thereby diminishing overall satisfaction with public services (De Gennaro et al., 2020).

Given citizens’ high regard for empathetic communication from frontline civil servants, developing chatbots with empathy capabilities should be a priority for public service departments in the era of intelligent governance. Based on the social presence theory, service platforms can effectively embody a citizen-centric service philosophy via the integration of AI empathy, fostering a friendly interactive atmosphere that evokes positive emotional responses from citizens. This human-centered interaction model is also considered a key mechanism for driving continued use of government chatbots (Meng & Ju, 2021). Research indicates that citizens interacting with chatbots exhibit clear expectations for service etiquette and emotional responsiveness, mirroring their interactions with real people (De Gennaro et al., 2020). If chatbots fail to meet these emotional needs, they are perceived as impolite or even disrespectful, negatively impacting service retention (Choi et al., 2021). Furthermore, the ability to express empathy helps mitigate negative emotions arising from service failures in government chatbots, reducing citizens’ frustration and thereby enhancing their willingness to continue using the chatbot (M. Li & Wang, 2023). Based on this, the following research hypotheses are proposed:

**H1.**  *Government chatbot empathy positively influences citizens’ willingness to continue using the service.*

### 2.4. The Mediating Role of Psychological Engagement and Information Richness

The Computers are Social Actors Theory holds that humans often unconsciously draw on the rules of interpersonal communication when interacting with computer-mediated platforms, perceiving such systems as social actors with inherent social attributes (H. Fan et al., 2022). Social presence theory further indicates that when individuals interact with intelligent computer media, specific mental models are often activated, generating psychological engagement. This is due to the fact that these forms of media can simulate real-world social contexts, prompting individuals to actively engage in interactions and maintain positive relationships (Juquelier et al., 2025; G. Li et al., 2024). Building on this, existing research demonstrates that enhancing the social characteristics of AI media (e.g., human-like behaviors) can effectively elevate users’ psychological engagement levels. For instance, Zhou et al. (2025) found that chatbots exhibiting human-like traits during service interactions significantly enhanced users’ psychological engagement and trust; this positive effect was even more pronounced when government chatbots demonstrated understanding of users’ emotions during conversations. Evidently, when government chatbots employ empathy strategies, human–robot interaction can more closely resemble interpersonal interactions. The authenticity and immediacy fostered by such experiences serve as the core mechanism driving the activation of users’ psychological engagement (X. Li & Wang, 2024). Simultaneously, an elevated level of psychological engagement helps optimize the interactive experience between citizens and government chatbots, leading citizens to perceive that their demands are valued and thus enhancing their overall satisfaction with public services. According to Expectation-Confirmation Theory, service satisfaction serves as a crucial antecedent variable influencing the intention to continue using the service—higher satisfaction correlates with stronger continued usage intent (Y. Park et al., 2024). Furthermore, as psychological engagement deepens, citizens may gradually develop behavioral habits of accessing government information and services through government chatbots, fostering dependency on these chatbot-based government services. Once this reliance is established, citizens become significantly less likely to abandon use, and their willingness to continue using the service increases accordingly. In summary, the following hypothesis is proposed:

**H2.**  *Psychological engagement mediates the effect of government chatbot empathy on citizens’ continued usage intention.*

Based on social presence theory, technological media provide individuals with diverse interactive channels, enabling them to access not only social information but also task-related functional information. In technology-mediated government service delivery, citizens particularly value the richness of the information they receive to swiftly address their current and potential service needs (C. Wang et al., 2021). Conversely, inadequate information delivery often compels users to resort to manual assistance, thus defeating the original purpose of technological solutions to boost efficiency and automation, a perception that users hold as a “disappointing technological experience” (Holloway & Beatty, 2008). Information richness stands as a core dimension for evaluating the information delivery efficacy of technological media, which is primarily reflected in the comprehensiveness of information content, the minimization of ambiguous information, and the timeliness of feedback (J. Xu et al., 2013). Users’ perception of information richness typically depends on the attributes of the delivery tool. For instance, factors like personalized design and content update frequency on government websites significantly influence users’ subjective assessment of information richness (Ha & Im, 2012). In government chatbot services, empathy may emerge as a critical factor influencing information delivery. This is because empathetic capabilities enable government chatbots to recognize and respond to citizens’ behavioral preferences, personality traits, and emotional states, thereby achieving personalized dialogue customization. This user-profile-based content matching enhances the relevance, accuracy, and completeness of government information, ultimately elevating citizens’ overall assessment of information richness (G. Park et al., 2023). Furthermore, by integrating emotional vocabulary, tone simulation, and even emojis, chatbot empathy elevates information delivery beyond functional utility to incorporate an emotional dimension. This emotionally embedded information format enhances the perceptibility and approachability of government communications (De Gennaro et al., 2020). Since information richness effectively addresses citizens’ complex information needs, it fosters continued motivation for using government chatbots. More importantly, when government chatbots deliver diverse content and timely feedback, human–robot interaction becomes more substantive and interactive experiences grow more favorable, further fostering long-term usage tendencies. Therefore, we propose the following hypothesis:

**H3.**  *Information richness mediates the effect of government chatbot empathy on citizens’ continued usage intention.*

### 2.5. The Regulatory Role of Exogenous and Endogenous Time Pressure

Time pressure refers to the perceived time constraints individuals experience when considering information, making decisions, or performing specific tasks (Ostrom et al., 2021). Current scholarship on time pressure primarily focuses on three perspectives: the coping perspective, the stress intensity perspective, and the stressor nature perspective. Of these three, the first two emphasize exploring individuals’ response mechanisms to time pressure and the impact of its intensity on behavioral outcomes. Scholarly findings have yielded mixed results, revealing that time pressure and behavioral performance may be linked by positive, negative, or inverted U-shaped associations (Baer & Oldham, 2006; Ohly & Fritz, 2010). However, these studies have failed to fully address the endogenous attributes of time pressure. On the contrary, the stressor nature perspective seeks to reveal the internal mechanisms through which time pressure influences individual behavior by exploring its core characteristics. Typical studies like X. Liu et al. (2017) propose that time scarcity arising from challenging factors such as task urgency and complexity constitutes challenge-based time pressure. Meanwhile, Chong et al. (2011) propose that inefficient communication and unreasonable task allocation can passively encroach on an individual’s time, giving rise to obstruction-based time pressure. Despite the theoretical expansion of time pressure classification boundaries achieved by these studies, their analytical focus is still limited to the field of work tasks. In practice, the formation of time pressure is not only constrained by objective task factors but may also be influenced by subjective individual traits such as time perception and efficiency worship. This occurs because individuals tend to desire completing specific tasks in less time, thereby allocating more time resources to matters they perceive as more important (Ostrom et al., 2021).

This paper draws upon the research framework of Hunt and Sandhu (2017) to categorize the time pressure perceived by citizens during government service processes into two types: exogenous time pressure and endogenous time pressure. Exogenous time pressure refers to the urgency citizens perception when handling government affairs due to explicit deadlines imposed by the service itself. This pressure stems from objective time constraints inherent to the task, compelling individuals to focus on rapidly and efficiently obtaining task-related information and enhancing communication with civil servants to complete the process. Consequently, it is characterized by low autonomy and high passivity. In contrast, endogenous time pressure arises from citizens’ tension regarding time resources due to personal traits such as their own sense of time and efficiency preferences. This pressure does not stem from exogenously imposed time constraints but rather from psychological perceptions generated during the autonomous allocation of time resources, exhibiting a high degree of autonomy.

Under exogenous time pressure, individuals interacting with government chatbots tend to de-emphasize the social aspects of human–robot interaction and prioritize the efficient acquisition of task-related information. At this point, citizens face a “dual pressure”: on the one hand, they possess a strong motivation to quickly obtain the information required to complete the task; on the other hand, objective time constraints limit their available cognitive resources, making it difficult to process and integrate additional information. Consequently, citizens under exogenous time pressure are more likely to focus on task completion than on the relational aspects of the interaction.

From the perspective of psychological engagement, government chatbot empathy may lose part of its value under exogenous time pressure. According to social presence theory, when citizens experience strong external time constraints, efficiency becomes more important than social connection, and users tend to favor less socially oriented technologies (Straub & Karahanna, 1998). In this context, empathic responses, such as expressions of understanding and emotional support, may be viewed as unnecessary conversational elements that distract attention from the primary goal of completing the service process. Therefore, exogenous time pressure is expected to weaken the positive effect of government chatbot empathy on psychological engagement.

From the perspective of perceived information richness, citizens under exogenous time pressure tend to prefer clear, explicit, and task-relevant information. Although empathic responses may enrich the social dimension of the interaction, they may simultaneously increase the amount of information that citizens must process within a limited period. Consequently, social cues conveyed through chatbot empathy may be perceived as cognitive interference rather than useful information, thereby reducing users’ evaluations of information richness (Pieters & Warlop, 1999). Consistent with this logic, X. Xu and Liu (2022) found that time pressure diminished users’ positive responses to chatbot humor. Therefore, exogenous time pressure is expected to weaken the positive effect of government chatbot empathy on perceived information richness.

Accordingly, we propose:

**H4a.**  *Exogenous time pressure negatively moderates the effect of government chatbot empathy on psychological engagement.*

**H4b.**  *Exogenous time pressure negatively moderates the effect of government chatbot empathy on perceived information richness.*

In contrast, under endogenous time pressure, individuals’ perceptions of time scarcity are primarily driven by their subjective sense of time and efficiency preferences rather than externally imposed deadlines (Ostrom et al., 2021). As a result, information-seeking behavior under endogenous time pressure is characterized by greater autonomy, more diverse objectives, and deeper information processing. Unlike the “urgent search” associated with exogenous time pressure, information seeking in this context is more likely to take the form of “exploratory processing”.

From the perspective of psychological engagement, government chatbot empathy is unlikely to be perceived as disruptive because citizens retain control over the pace of information processing. Instead, empathic responses may reduce the psychological cost of information acquisition, establish cognitive security, and foster a stronger sense of social presence. Consequently, citizens may become more willing to engage actively with the chatbot and devote greater attention to the interaction process. Therefore, endogenous time pressure is expected to strengthen the positive effect of government chatbot empathy on psychological engagement.

From the perspective of perceived information richness, government chatbot empathy may encourage citizens to ask more questions, explore available information more deeply, and seek a more comprehensive understanding of government services. Without intense external pressure, users are more likely to appreciate both the emotional support and informational value embedded in empathic communication. Previous studies have shown that in contexts characterized by relatively low stress and open interaction environments, users tend to pursue both emotional support and information comprehension when interacting with intelligent technologies (Juquelier et al., 2025; G. Park et al., 2023). Therefore, endogenous time pressure is expected to strengthen the positive effect of government chatbot empathy on perceived information richness.

Accordingly, we propose:

**H4c.**  *Endogenous time pressure positively moderates the effect of government chatbot empathy on psychological engagement.*

**H4d.**  *Endogenous time pressure positively moderates the effect of government chatbot empathy on perceived information richness.*

**Figure 1:**
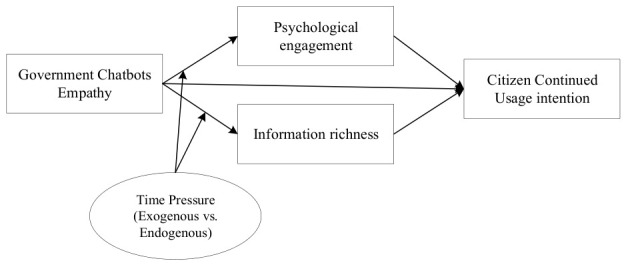
Research model.

In summary, the research model of this study is illustrated in Figure 1.

## 3. Empirical Research

Data were collected by using scenario experiment method. The scenario experiment method allows researchers to manipulate independent variables by placing participants in different decision-making scenarios. By comparing participants’ responses to varying levels of independent variables, causal relationships between independent and dependent variables can be identified, effectively balancing endogenous and exogenous validity in research (Qin & Li, 2020). In leading international public administration journals, scenario experiment method is also among the most frequently used experimental methods in behavioral public administration research (G. L. Chen et al., 2023). To minimize common method bias, researchers employed randomization techniques in each experiment, ensuring that the items presented to each participant were randomly assigned. Experiment 1 aimed to test the direct effect of government chatbot empathy on citizens’ continued usage intention, along with the parallel mediating effects of psychological engagement and information richness, thereby testing H1–H3. Experiment 2 sought to explore the moderating roles of exogenous and endogenous time pressure, thereby testing H4a–H4d.

### 3.1. Experiment 1

#### 3.1.1. Experimental Design

The demographic characteristics of the experimental sample are presented in Table 2. Given that the research objective focused on continued intention to use government chatbots, a sample composed of younger individuals with higher educational attainment is acceptable (X. Li & Wang, 2024).

A single-factor between-subjects design was adopted in this experiment, which recruited 148 participants and randomly assigned them to either the control group or the experimental group. The control group exhibited high chatbot empathy, while the experimental group demonstrated low chatbot empathy. To ensure data quality, the Credamo platform was utilized for sample collection. After excluding 16 invalid questionnaires that failed the attention check item from the initial sample, 148 valid samples were ultimately obtained. The demographic characteristics of the participants are presented in Table 1. During the experiment, participants were informed that their ID cards were about to expire and needed to be replaced promptly. Subsequently, participants were asked to imagine browsing a fictional government service platform website and interacting with a government chatbot to obtain information about renewing an expired ID card. To enhance participants’ differentiated perception of the chatbot’s intelligence, the experiment invited them to view simulated chat dialogue script screenshots and mentally place themselves within the conversational scenario.

Significant differences exist in how government chatbots interact with citizens between the “low chatbot empathy” group and the “high chatbot empathy” group. To manipulate chatbot empathy levels, human–robot dialogue scripts were designed based on research procedures from computer science regarding empathy communication encoding systems (Suchman et al., 1997), while referencing real conversation corpora from Beijing’s “Jingjing” and Shanghai’s “Xiao i”. Specifically, the high-empathy group’s chatbot demonstrated understanding and concern for citizens’ situations through “putting themselves in others’ shoes”. It proactively acknowledged users’ urgent needs and provided more emotionally resonant responses (e.g., “I understand you may need your ID urgently”) to simulate perspective-taking. We also incorporated emoticons to enhance emotional contagion and amplify affective expression. In contrast, the low-empathy expression group’s chatbot responses were more procedural, primarily focused on task completion. Its language style lacked responsiveness to user emotions, exhibiting lower emotional adaptability (e.g., “Please log in to the ‘National Government Service Platform’ or your local public security app, click ‘Document Processing’ → ‘ID Card Replacement’)”. The dialogue scripts are detailed in Table 3.

Finally, participants were asked to complete questionnaires measuring perceived chatbot empathy, willingness to continue using the service, and personal information. Drawing from Liu-Thompkins et al. (2022), the items measuring empathy in government chatbots were: “The chatbot tried to understand me better,” “The chatbot tried to understand my emotions,” and “The chatbot easily influenced my emotions”. Drawing from H. Fan et al.’s (2022) scale, items measuring willingness to continue using the government chatbot include: “In my work, I will consistently try to use the government chatbot” and “I am willing to use the government chatbot in the future”. Based on Bifulco et al. (2003), the items measuring psychological engagement were: “During interactions with the chatbot, I feel as if I am conversing face-to-face with a real person,” “I am highly emotionally and attentively engaged during interactions,” and “I feel supported by the other party during interactions”. Based on H. Kim and Niehm (2009), items measuring information richness include: “The chatbot provided detailed information during the interaction,” “The chatbot provided timely information during the interaction,” “The chatbot provided information I needed during the interaction,” and “The chatbot provided high-quality information during the interaction”. All scales employ a 7-point Likert scale, where 7 indicates “strongly agree” and 1 indicates “strongly disagree.”

#### 3.1.2. Experimental Results

Reliability Analysis. We used SPSS 22 to evaluate the reliability of our measurements. Reliability analysis yielded Cronbach’s alpha coefficients of 0.786, 0.803, 0.835, and 0.875 for government chatbot empathy, willingness to continue service usage, psychological engagement, and perceived information richness, respectively. All coefficients met the acceptable threshold for internal consistency, confirming the reliability of the four constructs (government chatbot empathy, citizens’ willingness to continue using government chatbot services, psychological engagement, and perceived information richness) in the present study.

Manipulation Check. To evaluate the effectiveness of government chatbot empathy manipulation, an independent-samples *t*-test was performed. Table 4 summarizes the results of the independent *t*-test analysis. The results revealed a significant difference in participants’ perceived empathy levels toward the government chatbot between the low-empathy and high-empathy groups (*p* < 0.001). Specifically, participants in the low-empathy group reported a mean score of 4.221 (SD = 1.433), while those in the high-empathy group achieved a mean score of 5.884 (SD = 1.067). Collectively, these findings confirm that the government chatbot empathy manipulation in the experiment was successful.

To examine the direct effect of government chatbot empathy on citizens’ willingness to continue service usage (Hypothesis 1), an independent-samples *t*-test was performed on the data of the two experimental groups. Table 5 summarizes the independent-samples *t*-test results for the effect of chatbot empathy on continued usage intention. The results showed a significant difference in willingness scores between the groups (*p* < 0.001): the low-empathy group had a mean score of 3.327 (SD = 1.797), while the high-empathy group obtained a mean score of 5.642 (SD = 1.318). Participants in the high-empathy group exhibited significantly greater willingness to continue using government chatbot services than those in the low-empathy group, thus supporting Hypothesis 1.

The parallel mediating effects of psychological engagement and perceived information richness on the relationship between government chatbot empathy and citizens’ continued usage intention were tested using Model 4 of the Process macro for SPSS. In this mediation model, as detailed in Table 6, government chatbot empathy was the independent variable, psychological engagement and perceived information richness served as parallel mediating variables, and continued usage intention was the dependent variable. Results indicated that the total effect of government chatbot empathy on continued usage intention was significant (*β* = 1.327, SE = 0.198, *p* < 0.001), whereas the direct effect of government chatbot empathy on continued usage intention was non-significant (*β* = 0.150, SE = 0.252, *p* = 0.552). The mediating effect of psychological engagement was significant (*β* = 0.500, SE = 0.141, 95% CI [0.240, 0.790]), and the mediating effect of perceived information richness was also significant (*β* = 0.677, SE = 0.142, 95% CI [0.410, 0.960]). These findings demonstrate that government chatbot empathy exerts an indirect influence on citizens’ continued usage intention through the dual parallel mediators of psychological engagement and perceived information richness: citizens perceive higher levels of psychological engagement and information richness when interacting with government chatbots with high (vs. low) empathy, which in turn enhances their intention to continue using the service. Hypotheses 2 and 3 are therefore supported.

#### 3.1.3. Discussion

Experiment 1 provides initial evidence for the positive effect of government chatbot empathy on citizens’ continued usage intention, extending prior research on artificial empathy (Liu-Thompkins et al., 2022) into the public service domain. Using a scenario-based experimental design, we manipulated chatbot empathy (low vs. high) in the context of government service inquiries. Consistent with our hypothesis, results showed that a high-empathy (vs. low-empathy) government chatbot significantly increased citizens’ willingness to continue using the service. This finding conceptually replicates the empathy–satisfaction linkage reported in commercial settings (e.g., travel insurance, rental services) and suggests that artificial empathy also enhances user retention in government–citizen digital interactions.

Building on social presence theory (Short et al., 1976; Biocca et al., 2003), we further examined the parallel mediating roles of psychological engagement and perceived information richness. The bootstrap mediation analysis (PROCESS Model 4) revealed that government chatbot empathy indirectly influenced continued usage intention through both psychological engagement and perceived information richness, with the direct effect becoming non-significant when mediators were included. These results indicate that when citizens interact with an empathic (vs. mechanical) government chatbot, they experience heightened psychological engagement and perceive the information provided as richer, which in turn fosters stronger intentions to continue using the chatbot service. Thus, Hypotheses 2 and 3 are supported.

Taken together, Study 1 demonstrates that artificial empathy in government chatbots not only directly enhances citizens’ continued usage intention but also operates through the dual mechanisms of psychological engagement and perceived information richness. These findings respond to recent calls for research on citizens’ responses to affective AI in public service encounters (Huang & Rust, 2021) and highlight the importance of designing empathetic conversational agents in government digital platforms. However, Study 1 does not examine potential boundary conditions. Drawing on social presence theory’s premise that contextual factors moderate users’ information processing (Biocca et al., 2003), we propose that the effectiveness of empathic government chatbots may depend on situational characteristics such as time pressure. Study 2 addresses this gap by investigating whether time pressure moderates the indirect effect of chatbot empathy on continued usage intention.

### 3.2. Experiment 2

#### 3.2.1. Experimental Design

The demographic characteristics of the experimental sample are shown in Table 1. We excluded samples that failed the attention check, resulting in a final sample size of 120 participants. A 2 (Government Chatbot Empathy: High empathy/Low empathy) × 2 (Time Pressure: Endogenous Time Pressure/Exogenous Time Pressure) experimental design was employed. Participants were randomly assigned to four groups, each comprising 30 individuals. The manipulation of government chatbot empathy remained identical to Experiment 1. The key difference was that participants in the two exogenous time pressure groups were informed they had only 5 min to complete the ID card replacement service, with any time-out resulting in a “service failure.” In contrast, participants in the two endogenous time pressure groups were advised to maintain good self-control over their time to avoid service delays due to procrastination. Subsequently, participants were invited to view the human–robot dialogue script used in Experiment 1 and imagine themselves as the service user in the conversation.

In addition to the scales used in Experiment 1, Experiment 2 introduced two additional scales: endogenous time pressure and exogenous time pressure. Drawing from scales developed by Cavanaugh et al. (2000) and Zapf (1993) regarding multi-source time pressure, the endogenous time pressure scale included items such as: “I am highly sensitive to the time I spend handling tasks,” “I tend to complete tasks in the shortest possible time,” and “When handling tasks, I often feel anxious about wasting time.” The exogenous time pressure scale includes items such as: “Fixed deadlines for task completion cause me stress,” “When facing clear deadlines, I tend to focus my attention on completing the task itself,” and “Objective time constraints compel me to gather necessary information and advance tasks at a faster pace.” All scales were measured using a seven-point Likert scale, where seven indicates “strongly agree” and one indicates “strongly disagree”.

#### 3.2.2. Experimental Results

Reliability Analysis. Reliability analysis yielded Cronbach’s alpha coefficients of 0.775, 0.781, 0.862, 0.826, 0.891, and 0.854 for government chatbot empathy, continued usage intention, psychological engagement, information richness, endogenous time pressure, and exogenous time pressure, respectively. All coefficients met the acceptable threshold for internal consistency, confirming the reliability of the six constructs (government chatbot empathy, continued usage intention, psychological engagement, information richness, endogenous time pressure, and exogenous time pressure) in the present study.

Manipulation Check. Independent samples *t*-tests revealed a significant difference in participants’ perceived government chatbot empathy levels between the low- and high-empathy groups (*p* < 0.001). Table 7 summarizes the results of independent-samples *t*-tests for experimental manipulations of empathy and time pressure. Specifically, the low-empathy group reported a mean score of 4.114 (SD = 1.397), while the high-empathy group had a mean score of 5.752 (SD = 1.106). Manipulation of time pressure also yielded the expected results: the exogenous time pressure group had a mean score of 6.214 (SD = 1.367, *p* < 0.001), and the endogenous time pressure group a mean score of 2.973 (SD = 1.549, *p* < 0.001). These findings verify the validity of the experimental manipulations for both government chatbot empathy and time pressure.

Testing Hypotheses 4a–4d. As shown in Table 8 and Table 9, we ran a general linear model in SPSS 22 using composite variables, followed by examining the simple slopes of all significant interactions. For psychological engagement, the interaction between government chatbot empathy and exogenous time pressure was significantly negative (*β* = −0.458, SE = 0.132, 95% CI [−0.717, −0.199], *p* < 0.001). Simple slope analysis revealed that under exogenous time pressure, the effect of government chatbot empathy on psychological engagement was non-significant (*β* = −0.112, SE = 0.205, 95% CI [−0.514, 0.290], *p* = 0.586), whereas under endogenous time pressure, the effect was significantly positive (*β* = 0.768, SE = 0.172, 95% CI [0.431, 1.105], *p* < 0.001). The interaction with endogenous time pressure was also significant (*β* = 0.186, SE = 0.094, 95% CI [0.002, 0.370], *p* < 0.05). These results support H4a (exogenous time pressure negatively moderates the empathy–psychological engagement link) and H4c (endogenous time pressure positively moderates this link). In other words, when citizens face externally imposed deadlines, the positive impact of chatbot empathy on psychological engagement is eliminated; when they experience self-imposed time concerns, the positive impact is amplified.

As shown in Table 9 and Table 10, for perceived information richness, the interaction between government chatbot empathy and exogenous time pressure was significantly negative (*β* = −0.152, SE = 0.065, 95% CI [−0.279, −0.025], *p* < 0.05). Simple slope analysis indicated that under exogenous time pressure, the effect of government chatbot empathy on perceived information richness was non-significant (*β* = 0.694, SE = 0.999, 95% CI [−1.264, 2.652], *p* = 0.487), whereas under endogenous time pressure, the effect was significantly positive (*β* = 0.796, SE = 0.159, 95% CI [0.484, 1.108], *p* < 0.001). The interaction with endogenous time pressure was significantly positive (*β* = 0.214, SE = 0.081, 95% CI [0.055, 0.373], *p* < 0.01). These results support H4b (exogenous time pressure negatively moderates the empathy–information richness link) and H4d (endogenous time pressure positively moderates this link). Collectively, these findings confirm the double-edged sword effect of government chatbot empathy on citizens’ continued usage intention. Accordingly, Hypotheses 4a–4d are all supported.

#### 3.2.3. Discussion

Experiment 2 extends the findings of Experiment 1 by examining a critical boundary condition—time pressure—under which the beneficial effects of government chatbot empathy on citizens’ continued usage intention may be attenuated. Building on the premise of social presence theory that contextual factors moderate users’ information processing and responses in computer-mediated interactions (Biocca et al., 2003), we distinguished between endogenous time pressure (self-imposed, procrastination-related) and exogenous time pressure (externally imposed, fixed deadlines). The results revealed a double-edged sword effect of government chatbot empathy.

Specifically, under endogenous time pressure, government chatbot empathy continued to exert a significant positive effect on both psychological engagement and perceived information richness, replicating the pattern observed in Experiment 1. However, under exogenous time pressure, the effect of government chatbot empathy became non-significant for both psychological engagement and perceived information richness. These findings support Hypotheses 4a–4d and demonstrate that the effectiveness of empathic government chatbots is not universal but critically depends on the source and nature of time constraints.

From a theoretical standpoint, exogenous time pressure imposes a rigid, scarce temporal resource that forces citizens to prioritize task completion speed over relational or affective considerations. In such contexts, empathic responses may be perceived as less relevant to users’ immediate task goals, thereby attenuating the positive influence of government chatbot empathy on psychological engagement and perceived information richness. As a result, the beneficial effects observed under endogenous time pressure become non-significant under exogenous time pressure. Conversely, endogenous time pressure arises from an individual’s own sensitivity to time use and procrastination avoidance, which does not conflict with the relational benefits of empathy; rather, empathic chatbot behavior may still foster engagement and enrich information processing, as citizens retain a sense of control over their pace.

Moreover, Experiment 2 provides evidence against the unconditional positive effect of artificial empathy in government service settings. While prior research has largely emphasized the benefits of empathic chatbots (Liu-Thompkins et al., 2022; Huang & Rust, 2021), our findings reveal a critical contingency: when citizens face externally imposed deadlines, empathic chatbots can become undesirable. This result resonates with recent work on the dark side of artificial empathy and responds to calls for investigating when feeling AI might backfire (G. Li et al., 2024).

Practically, for government agencies deploying chatbots for time-sensitive services (e.g., ID card renewal with legal deadlines), a high-empathy design may reduce rather than enhance continued usage intention. In such scenarios, a more task-focused, neutral chatbot style might be preferable. Conversely, for self-paced services where citizens control their own time (e.g., information inquiry or form preparation), empathic chatbots remain effective.

Taken together, Experiment 2 not only replicates the mediating roles of psychological engagement and perceived information richness established in Experiment 1 but also delineates an important boundary condition. The interaction between artificial empathy and time pressure advances our understanding of when and for whom empathetic government chatbots enhance or undermine citizen experiences in digital public service encounters.

## 4. General Discussion

### 4.1. Theoretical Implications

Although chatbot empathy is recognized for their potential to bridge the emotional gap between humans and AI in human–robot interaction (Liu-Thompkins et al., 2022), empirical research on government chatbot empathy remains scarce. By integrating fundamental premises of social presence theory (Short et al., 1976; Biocca et al., 2003), this study fills this research gap by systematically examining whether, how, and under what conditions government chatbot empathy influences citizens’ willingness to continue using them. By adopting research approach, this study actively responds to the recent scholarly call for advancing research on citizens’ psychological responses to emotional intelligence (Huang & Rust, 2021) and makes theoretical contributions in the following three aspects.

First, only a few studies have preliminarily explored the impact of government chatbot empathy on user behavioral responses, and conclusions in related research are inconsistent (G. Park et al., 2023; Huang & Rust, 2021). Moreover, embedding chatbot empathy within government contexts represents an emerging phenomenon, lacking systematic empirical validation and theoretical expansion (Huang & Rust, 2021; Adler et al., 2023). Consequently, this study focuses on the public domain of government services, systematically analyzing the double-edged sword effect of government chatbot empathy on citizens’ continued usage intention. It introduces psychological engagement and information richness as parallel mediating variables while accounting for the moderating effects of multi-source time pressure. Building on this foundation, this study adopts a scenario experimental design to enhance the credibility of causal inference, expands theoretical insights into the efficacy of emotional intelligence, and provides empirical evidence with higher external validity for the applicability boundaries and operational mechanisms underlying the “double-edged sword effect” of government chatbot empathy.

Secondly, existing research on users’ response mechanisms to government chatbot empathy primarily relies on the “computer as a social actor” paradigm, emphasizing the role of users’ social motivations in the interaction process. Building upon this foundation, this paper introduces social presence theory, positing that citizens in human–robot interaction scenarios for government services will evaluate both the psychological engagement elicited by government chatbot empathy and the information richness conveyed by government chatbots. Through the integration of parallel mediating mechanisms related to psychological engagement and information richness, this study constructs an integrated effectiveness analysis framework for government chatbot empathy that integrates social and functional attributes, thereby systematically revealing the underlying mechanisms through which they influence citizens’ continued usage intention. Additionally, taking inspiration from recent advancements in the Feeling AI (Huang & Rust, 2021), this research offers insights for the application of emotional intelligence technologies in public services. Meanwhile, this study extends the application of social presence theory from traditional contexts like education, marketing, and organizational behavior to government service scenarios, providing empirical evidence for its applicability in the emerging field of intelligent human–robot interaction in government services. In addition, this study establishes a closer connection between government chatbot research and emerging AI-based cognitive assessment research. Specifically, the constructs of psychological engagement and perceived information richness can be understood as important cognitive and evaluative responses formed during human–AI interaction. By examining how citizens cognitively evaluate empathetic government chatbots under different situational conditions, this study provides empirical insights into how AI systems may shape the measurement and assessment of users’ psychological and behavioral responses in digital governance environments.

Finally, this study validates the boundary conditions under which government chatbot empathy positively influence citizens’ willingness to continue using them, revealing the operational mechanisms underlying “double-edged sword” effect. The results of this study indicate that exogenous time pressure significantly attenuates the positive impact of government chatbot empathy on perceptions of psychological engagement and information richness, whereby the mediating pathways became non-significant. In contrast, endogenous time pressure exerts no such moderating effect. This finding aligns with the core tenet of the social presence theory, which posits that individuals’ information processing patterns are shaped by the media environments in which they are situated. The empirical findings further uncover that when objective time constraints driven by exogenous systems or processes exist in government service delivery, government chatbot empathy may instead trigger negative reactions such as information delays and emotional burden. Empirical finding complements existing research primarily focused on chatbot anthropomorphism, technical characteristics, and identity disclosure (G. Park et al., 2023; J. Fan et al., 2023). More importantly, it addresses ongoing debates regarding the cross-context applicability of emotional intelligence. As Castelo et al. (2023) noted, users do not maintain a consistent positive attitude toward service robots with empathy capabilities. Empirical validation is provided for the applicability of Castelo’s perspective in the public service context, prompting researchers and practitioners to carefully assess the application scenarios and triggering conditions of government chatbot empathy and avoid the blind pursuit of emotional intelligence technologies. In addition, although the present study focuses on government service interactions, the underlying mechanisms it uncovers—namely, how AI empathy interacts with user engagement, perceived information quality, and time pressure to shape continued usage—may also inform the application of AI in cognitive assessment. Cognitive assessment tasks, whether administered by human or AI, are inherently dependent on the assessee’s psychological engagement, perceived clarity of presented information, and sensitivity to time constraints. Our findings suggest that embedding empathy into AI-driven assessment interfaces could enhance user cooperation and data completeness under self-paced conditions, yet might backfire under strict time limits by increasing perceived burden or distraction. As such, this research offers initial theoretical groundwork for designing emotionally calibrated AI systems that support reliable measurement of cognitive responses.

### 4.2. Practice Implications

Based on the research findings, two practice implications are proposed, although it should be noted that these findings are derived from scenario-based experiments and thus warrant further validation in real-world government service settings.

First, government departments generally tend to deploy mechanistic chatbots with low-empathy capabilities to handle brief, one-off administrative service delivery tasks (Nißen et al., 2022). However, research by Castelo et al. (2023) has shown that chatbots lacking emotional responsiveness can elicit user frustration and even erode overall service satisfaction. The present study provides experimental evidence that, within a simulated government service context, higher levels of government chatbot empathy are associated with stronger continued usage intention among citizens. Accordingly, government departments may consider empathy as a potentially important design element in robotic service delivery and explore empathy-oriented chatbot design as one possible strategy for enhancing citizens’ willingness to continue using government chatbot services. From a technological perspective, several potential pathways could be considered for implementing government chatbot empathy: the embedding of fixed emotional scripts (Leite et al., 2013), the construction of system architectures with continuous learning capabilities (Lim & Okuno, 2015), and the supplementation of such capabilities with multimodal perception and analysis technologies (Juquelier et al., 2025). At the human–robot dialogue level, users’ perceived effectiveness of empathy in government chatbots might be enhanced through such strategies as identifying citizens’ background information to generate personalized responses, simulating perspective-taking, using emoticons to convey emotional cues, and incorporating emotionally resonant linguistic expressions such as “Don’t worry” and “I understand you.” However, these recommendations should be viewed as potential applications inferred from the experimental findings rather than practices that have already been validated in operational government service environments. Future field studies are needed to assess their effectiveness in real-world contexts.

Second, the experimental findings suggest that the effectiveness of government chatbot empathy may vary according to citizens’ perceived time pressure. Therefore, government chatbot systems could potentially benefit from adaptive interaction strategies that account for different time-pressure conditions. In order to achieve dynamic synergy between public services and user needs, government chatbots could be designed to integrate advanced AI algorithms—including natural language processing and affective computing—from the outset of human–robot interactions. Such algorithms, in principle, could analyze emotional vocabulary, speech rate fluctuations, expression patterns, and keyword utilization in users’ linguistic input, thereby helping to identify citizens’ time pressure types (e.g., exogenous vs. endogenous time pressure). Based on such analysis, the chatbot system might dynamically adjust its linguistic style, response speed, and level of empathy. For instance, when a user experiencing exogenous time pressure is identified, the system could prioritize concise, low-empathy, result-oriented responses in an attempt to reduce user wait times and cognitive load. Conversely, when detecting users under endogenous time pressure, the chatbot might appropriately enhance its empathy through active listening and emotional responses to strengthen its empathy communication. Because these recommendations are derived from a scenario-based experiment, their practical feasibility and effectiveness should be further examined through pilot implementations and field evaluations before large-scale adoption.

## 5. Conclusions and Limitations

### 5.1. Conclusions

Based on social presence theory and employing a scenario experiment method, this study empirically analyzes the impact of government chatbot empathy on citizens’ willingness to continue using them. Key findings are as follows: First, within the experimental scenario, government chatbot empathy positively influences citizens’ willingness to continue using them. Second, government chatbot empathy indirectly influences such willingness through psychological engagement and perceived information richness, with the impact becoming more pronounced as the capability of government chatbot empathy increases. Third, exogenous time pressure weakens and neutralizes the positive effect of government chatbot empathy on citizens’ perceptions of psychological engagement and information richness; endogenous time pressure enhances this positive effect. In other words, government chatbot empathy exerts a double-edged sword effect on citizens’ willingness to continue using the service.

Overall, the findings indicate that AI-enabled government chatbots are not merely technical tools for service delivery, but also important contexts in which citizens’ cognitive perceptions and psychological responses are formed and evaluated. More specifically, the present study demonstrates these relationships within a controlled experimental setting and provides preliminary evidence regarding the mechanisms through which government chatbot empathy may influence citizens’ behavioral intentions. In AI-mediated assessment environments, citizens’ perceptions of engagement and information richness can significantly influence their behavioral judgments and continued interaction intentions. At the same time, the different effects of exogenous and endogenous time pressure further suggest that cognitive and behavioral responses to AI systems are dynamic and context-dependent. Nevertheless, caution is warranted when extending these findings beyond the experimental context. While the results suggest promising directions for the design of future government chatbot systems, additional research conducted in real government service settings is necessary to establish the generalizability and practical applicability of these findings. Therefore, while AI technologies provide new opportunities for understanding and assessing human cognition and behavior in digital governance settings, attention should also be paid to the situational factors that may shape or constrain these assessment outcomes.

### 5.2. Research Limitations

This study has the following three limitations. First, methodological constraints. The scenario-based experimental approach employed in this research enhances endogenous validity by minimizing interference from participants’ subjective experiences. However, this method may also limit the practical applicability of the findings. Future studies should consider adopting qualitative research methods (such as multi-case comparative analysis) to address this limitation. Second, research framework limitations. This study did not reveal the connection between the willingness to continue using government chatbots and their actual usage. In reality, there may be underlying mechanisms between acceptance and usage, such as perceived controllability and rational emotions. Therefore, this study can serve as a starting point, with future research focusing on investigating the underlying mechanisms between the government chatbot empathy and their actual usage. Third, subject limitations. This study identified the antecedent of continued usage intention for government chatbots—empathy—but did not further explore the antecedents of empathy itself. Future research will therefore comprehensively investigate the drivers of empathy in government chatbots across psychological, social, technological, and cultural dimensions.

## Figures and Tables

**Table 1:** Synthesis of prior research on artificial empathy in customer–chatbot service encounters.

Literature	Theory	Sector(s)	Social-Related Mediator(s)	Information Richness	Moderator(s)	Outcome(s)
B. Liu and Sundar (2018)	CASA	Healthcare	X		Customer-related	Overall evaluation
Adam et al. (2021)	CASA	Banking	X			User compliance
J. Fan et al. (2023)	Confirmation theory	Hospitality			Chatbot- and employee-related	Customer retention
Markovitch et al. (2024)	Attribution theory	Retail			Chatbot-related	Customer satisfaction
This study	Social presence theory	Public sector	X	X	Context-related (time pressure)	Citizens’ Continued Usage Intention

Note: X indicates that this content has been examined.

**Table 2:** Demographic characteristics of participants.

		Experiment 1	Experiment 2
Variable	Classification	*N* = 148	*N* = 120
Gender	female	72	52
	Male	76	68
Age	18–22	69	63
	23–27	30	32
	28–32	26	15
	≥33	23	10
Level of Education	Undergraduate	91	70
	Master’s degree	55	32
	Ph.D.	2	18

**Table 3:** Dialogue scripts between two types of government chatbots and citizens.

Low-Empathy Human–Computer Dialogue Script for Government Chatbot	High-Empathy Human–Computer Dialogue Script for Government Chatbots
Government Chatbot: Hello, I am the “Smart Service Assistant.” What service would you like to apply for? Citizen: I need to renew my ID card. My current one is about to expire. Government Chatbot: Please log in to the “National Government Service Platform” or your local public security app. Click “Document Processing” → “ID Card Renewal,” fill in the information, and upload a photo. Citizen: I’m currently not in my local area. Can I apply online? Government Chatbot: If your registered residence offers remote processing services, select “Remote Replacement.” Details at this link: [www.gov.cn/id-service] Citizen: What materials do I need to prepare? Government Chatbot: Prepare the following materials: 1. Your current ID card 2. A recent, regulation-compliant passport photo 3. A fully completed application form Citizen: How long will it take to receive it? Government Chatbot: Processing typically takes 15 business days. Please be patient. Government Chatbot: Any other questions? Citizen: No, thank you. Government Chatbot: Alright, have a pleasant day!	Government Chatbot: Hello, welcome to the “Government Service Assistant.” I’m Xiaozheng. How may I assist you today? Citizen: My ID card is about to expire, and I need to renew it. But I’ve been quite busy lately and am unsure about the process. Government Chatbot: I completely understand your situation. The renewal process isn’t complicated at all. Shall I walk you through it step by step? Citizen: Sure, thank you. Government Chatbot: You’re welcome 😊! Simply log in to the “National Government Service Platform” or your local government app, select “ID Card Replacement” under “Document Services,” and follow the prompts to fill out the form and upload your photo. Citizen: I’m not currently in my registered residence city. Can I still apply? Government Chatbot: Yes, many cities now support cross-city replacements. If your city offers this service, the system will automatically prompt “Cross-City Processing.” If you’re unsure, I can check the list of eligible cities for you. Citizen: How long does it take to process? Government Chatbot: I understand you may need your ID urgently. Typically, the document is produced within 15 business days and mailed to your selected address. Some cities offer expedited service—would you like me to check if it’s available? Citizen: No rush. What do I need to bring when applying? Government Chatbot: You only need to prepare your current ID card, standard-compliant recent photos (some regions offer on-site photo services), and complete the required information. Government Chatbot: If you encounter any difficulties during the process, feel free to click “Human Assistance” or call me again anytime—I’m always here. Citizen: That’s so thoughtful, thank you! Government Chatbot: You’re welcome. Wishing you a smooth ID renewal process and all the best! 🌟

**Table 4:** The results of the independent *t*-test analysis.

Groups	Samples	Mean	SD	*p* Value
Low-empathy group	74	4.221	1.433	<0.001
High-empathy group	74	5.884	1.067	<0.001

**Table 5:** Independent-Samples *t*-test Results for the Effect of Chatbot Empathy on Continued Usage Intention.

Variables		Mean	SD	*t* Value	Cohen’s *d*	*p* Value
Citizens’ continued usage intention	Low-empathy group	3.327	1.797	8.938	1.469	<0.001
	High-empathy group	5.642	1.318			

**Table 6:** The results of the mediation analysis.

Effect	*β*	SE	*p* Value	95% CI	
				Lower	Upper
Total effect (government chatbot empathy on continued usage intention)	1.327	0.198	<0.001	—	—
Direct effect (government chatbot empathy on continued usage intention)	0.150	0.252	>0.05	—	—
Indirect effect (government chatbot empathy on continued usage intention via psychological engagement)	0.501	0.141	<0.001	0.240	0.790
Indirect effect via (government chatbot empathy on continued usage intention via perceived information richness)	0.677	0.142	<0.001	0.410	0.960

**Table 7:** The results of independent-samples *t*-tests for experimental manipulations of empathy and time pressure.

Groups	Samples	Mean	SD	*p* Value
Low-empathy group	60	4.114	1.397	<0.001
High-empathy group	60	5.752	1.106	<0.001
Exogenous time pressure group	60	6.214	1.367	<0.001
Endogenous time pressure group	60	2.973	1.549	<0.001

**Table 8:** Moderation effect of time pressure in psychological engagement relationships.

Dependent Variable: Psychological Engagement	*β*	SE	*p* Value	95% CI	
				Lower	Upper
Exogenous time pressure	−3.201	0.724	<0.001	−4.632	−1.770
Government chatbot empathy	0.346	0.157	<0.05	0.038	0.654
Exogenous time pressure × Government chatbot empathy	−0.458	0.132	<0.001	−0.717	−0.199
Endogenous time pressure	0.127	0.109	0.247	−0.088	0.342
Government chatbot empathy	0.582	0.144	<0.001	0.299	0.865
Endogenous time pressure × Government chatbot empathy	0.186	0.094	<0.05	0.002	0.370

**Table 9:** Simple slope analysis of government chatbot empathy under different time pressure conditions.

Dependent Variable	Time Pressure Condition	*β*	SE	*p* Value	95% CI	
					Lower	Upper
Psychological engagement	Exogenous	−0.112	0.205	0.586	−0.514	0.290
	Endogenous	0.768	0.172	<0.001	0.431	1.105
Perceived information richness	Exogenous	0.694	0.999	0.487	−1.264	2.652
	Endogenous	0.796	0.159	<0.001	0.484	1.108

**Table 10:** Moderation effect of time pressure in perceived information richness.

Dependent Variable: Information Richness	*β*	SE	*p* Value	95% CI	
				Lower	Upper
Exogenous time pressure	−3.102	0.521	<0.001	−4.124	−2.080
Government chatbot empathy	0.846	0.997	=0.397	−1.108	2.800
Exogenous time pressure × Government chatbot empathy	−0.152	0.065	<0.05	−0.279	−0.025
Endogenous time pressure	1.524	0.168	<0.001	1.194	1.854
Government chatbot empathy	0.582	0.137	<0.001	0.313	0.851
Endogenous time pressure × Government chatbot empathy	0.214	0.081	<0.01	0.055	0.373

## Data Availability

The data supporting the findings of this study are available from the corresponding author upon reasonable request. The data are not publicly available due to privacy considerations.
